# Role of Artificial Intelligence in COVID-19 Detection

**DOI:** 10.3390/s21238045

**Published:** 2021-12-01

**Authors:** Anjan Gudigar, U Raghavendra, Sneha Nayak, Chui Ping Ooi, Wai Yee Chan, Mokshagna Rohit Gangavarapu, Chinmay Dharmik, Jyothi Samanth, Nahrizul Adib Kadri, Khairunnisa Hasikin, Prabal Datta Barua, Subrata Chakraborty, Edward J. Ciaccio, U. Rajendra Acharya

**Affiliations:** 1 Department of Instrumentation and Control Engineering, Manipal Institute of Technology, Manipal Academy of Higher Education, Manipal 576104, India; anjan.gudigar@manipal.edu (A.G.); sneha.nayak@manipal.edu (S.N.); rohit4gm@gmail.com (M.R.G.); chinmaydharmik@gmail.com (C.D.); 2 School of Science and Technology, Singapore University of Social Sciences, Singapore 599494, Singapore; cpooi@suss.edu.sg; 3 Department of Biomedical Imaging, Faculty of Medicine, University of Malaya, Kuala Lumpur 50603, Malaysia; waiyeec@ummc.edu.my; 4 Department of Cardiovascular Technology, Manipal College of Health Professions, Manipal Academy of Higher Education, Manipal 576104, India; samanth.jyothi@manipal.edu; 5 Department of Biomedical Engineering, Faculty of Engineering, University of Malaya, Kuala Lumpur 50603, Malaysia; nahrizuladib@um.edu.my (N.A.K.); khairunnisa@um.edu.my (K.H.); 6 Cogninet Brain Team, Cogninet Australia, Sydney, NSW 2010, Australia; Prabal.Barua@usq.edu.au; 7 School of Business (Information Systems), Faculty of Business, Education, Law & Arts, University of Southern Queensland, Toowoomba, QLD 4350, Australia; 8 Faculty of Engineering and Information Technology, University of Technology Sydney, Sydney, NSW 2007, Australia; subrata.chakraborty@uts.edu.au; 9 Faculty of Science, Agriculture, Business and Law, University of New England, Armidale, NSW 2351, Australia; 10 Department of Medicine, Columbia University Medical Center, New York, NY 10032, USA; ciaccio@columbia.edu; 11 School of Engineering, Ngee Ann Polytechnic, Singapore 599489, Singapore; Rajendra_Udyavara_ACHARYA@np.edu.sg; 12 Department of Biomedical Informatics and Medical Engineering, Asia University, Taichung 41354, Taiwan; 13 International Research Organization for Advanced Science and Technology (IROAST), Kumamoto University, Kumamoto 860-8555, Japan

**Keywords:** artificial intelligence, computer-aided diagnostic tool, deep neural networks, hand-crafted feature learning, supervised learning

## Abstract

The global pandemic of coronavirus disease (COVID-19) has caused millions of deaths and affected the livelihood of many more people. Early and rapid detection of COVID-19 is a challenging task for the medical community, but it is also crucial in stopping the spread of the SARS-CoV-2 virus. Prior substantiation of artificial intelligence (AI) in various fields of science has encouraged researchers to further address this problem. Various medical imaging modalities including X-ray, computed tomography (CT) and ultrasound (US) using AI techniques have greatly helped to curb the COVID-19 outbreak by assisting with early diagnosis. We carried out a systematic review on state-of-the-art AI techniques applied with X-ray, CT, and US images to detect COVID-19. In this paper, we discuss approaches used by various authors and the significance of these research efforts, the potential challenges, and future trends related to the implementation of an AI system for disease detection during the COVID-19 pandemic.

## 1. Introduction

COVID-19 was first reported by the Wuhan Municipal Health Commission, China, in December 2019. It is caused by the severe acute respiratory syndrome coronavirus 2 (SARS-CoV-2), and is considered one of the deadliest global pandemics in history [1]. The World Health Organization (WHO) declared the COVID-19 outbreak a pandemic in March 2020, and there have been 203,944,144 cases and 4,312,902 deaths globally according to the WHO statistics of 12 August 2021 (available online: https://covid19.who.int/table (accessed on 12 August 2021)). The pandemic situation has caused worldwide distress by affecting people socially, medically, and economically. This infectious disease in severe form often leads to acute respiratory syndrome and the development of pneumonia. The outbreak was thought to be initiated via zoonotic spread from the seafood markets in Wuhan, China. Later, it was believed that transmission between humans was responsible for community spread of the infection throughout the world, and approximately 200 countries have been affected by this pandemic [2,3,4,5]. Although individuals of all ages are at risk of being infected, severe COVID-19 symptoms are more likely in people aged 60 and above, and individuals with comorbidities.

**Figure 1:**
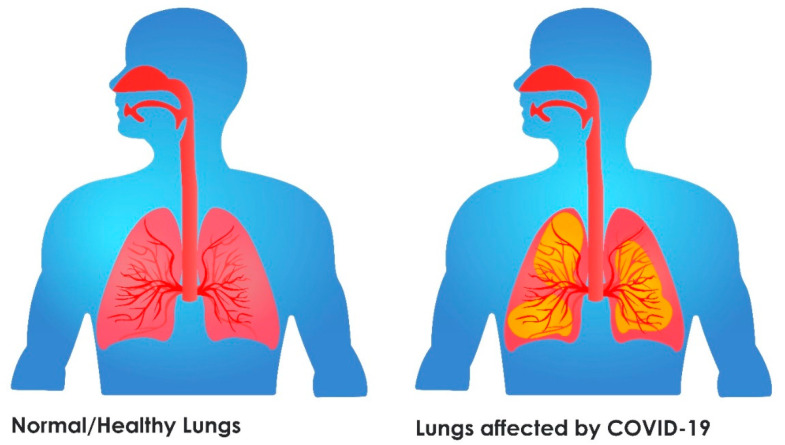
Pictorial representation of normal and COVID-19 affected lungs.

Once the SARS-CoV-2 virus enters the body via respiratory aerosol, it acts on the respiratory system, and affects patients with varying degrees of clinical severity. During the initial days of infection, the clinical presentation remains asymptomatic, although immune response is mediated in the body. Those persons affected are infectious at this phase, and the disease can be diagnosed by nasal swab [6,7,8]. Further migration of the virus from nasal epithelial cells into the upper respiratory tract results in symptoms of fever, dry cough, malaise, etc. The majority of infected patients do not progress beyond this phase, as the immune response from the host is sufficient to contain the disease from spreading to the lower respiratory tract and lungs [9] (refer to Figure 1).

Approximately one-fifth of infected cases develop lower respiratory tract infection, and these patients present with acute respiratory distress syndrome (ARDS). Histologically, this stage reveals lung sequestration along with host cell apoptosis. Persistent inflammation and diffuse alveolar damage are common histopathologic patterns observed among the infected patients exhibiting ARDS [5,10].

COVID-19 affects people in different ways. Asymptomatic patients will have positive nasal swab results and normal chest X-ray images. Patients with mild illness exhibit different commonly known symptoms such as fever, sore throat, dry cough, malaise and body aches or nausea, vomiting, abdominal pain, and loose stools. Patients with moderate illness show symptoms of pneumonia with no significant hypoxemia (persistent fever and cough). This group of infected patients also shows abnormal lesions on high-resolution chest computed tomography (CT). Severe illness is defined as patients who present with pneumonia and significant systemic hypoxemia (SpO_2_ < 92%). In cases of critical infection, the patients show life-threatening complications such as ARDS, along with shock, coagulation defects, encephalopathy, heart failure, and acute kidney injury [11,12,13,14,15,16,17].

Disease confirmation and the severity of the disease can be determined by nasal/throat swab, several serological tests, and imaging modalities. Reverse transcription polymerase chain reaction (RT-PCR) remains the best molecular method in the diagnosis of the disease [18]. However, as in the case of other diagnostic methods, RT-PCR is not error-free. It is time consuming, expensive, and requires manual effort. In order to judge the diagnostic test results accurately, expert clinicians are required to read them. The correct interpretation of the test results requires a high level of clinical expertise, as the data may vary significantly from person to person [19]. Moreover, the availability of test kits is limited, especially in rural geographical regions, and, if available, the quality of the kits may not be guaranteed. Moreover, persons can experience discomfort—slight pain and irritation—during the nasal swab test. Using image modalities such as X-rays or CT scans, it is possible to obtain a quick result for critical situations, even before receiving RT-PCR test results.

In order to overcome these existing shortcomings, many computer-aided diagnostic tools (CADTs) using artificial intelligence (AI) and machine learning techniques have been utilized to support clinical findings from the imaging modalities [20]. These prediction techniques can precisely detect the disease, thereby aiding in the prevention and detection of epidemiologic risk [21]. These automated tools using cost-effective imaging modalities assist to address COVID-19 by preventing false negative reports, and can be used in the case of scarcity or non-availability of RT-PCR test kits in rural areas. Researchers have reported exhaustive studies using imaging modalities for the detection of COVID-19 [19,21,22,23,24,25,26,27,28,29]. Although these review papers have shown the significance of deep learning and machine leaning algorithms for automated detection, this paper explores the following key points in association with detection:

1. The state-of-the-art AI techniques (deep neural network (DNN) and hand-crafted feature learning (HCFL) based models) used to detect COVID-19.
2. Analysis of the results of AI techniques with various imaging modalities.
3. The key challenges and future direction in the detection of COVID-19.

The structure of this paper is as follows. Section 2 describes the search criteria applied to accumulate and filter research articles from various sources. In Section 3, a consolidated review of extensively employed AI techniques using different medical imagery for COVID-19 detection is presented. The results using various datasets and methods are analyzed in Section 4. The key challenges, future scope, and recommendations are discussed in Section 5. Finally, the conclusions of our systematic review are presented in Section 6.

## 2. Search Criteria and Selection Process

The methodology from the Preferred Reporting Items for Systematic Reviews and Meta-Analyses (PRISMA) statement [30] was adopted in this study. The systematic search process was carried out using the search query on Scopus, Google Scholar, PubMed, and Springer. The following search items were used: “COVID-19”, “Automated detection of COVID-19”, “Computer-aided diagnosis for COVID-19”, “Deep learning techniques for COVID-19” (using “Chest X-ray” OR “Chest computed tomography” OR “Lung Ultrasound”), and “Database for COVID-19”. In order to widen the search process, we also included AI algorithms with specific techniques, its subfield, and its utilization (such as “CAD tools”, “Convolutional neural networks”, “Machine Learning”, “Classification”, and “Identification”). Related articles written in English from 2020 to 2021 were downloaded, not limited to country or region of the author. The search process took 22 days to complete. A total of 1277 articles were found. The relevance of the downloaded articles to the main aims of this study was verified using a search string strategy. Articles related to detection of COVID-19 using clinical data, statistical analysis, and case studies with no data mining and deep leaning techniques were excluded from the selection.

**Figure 2:**
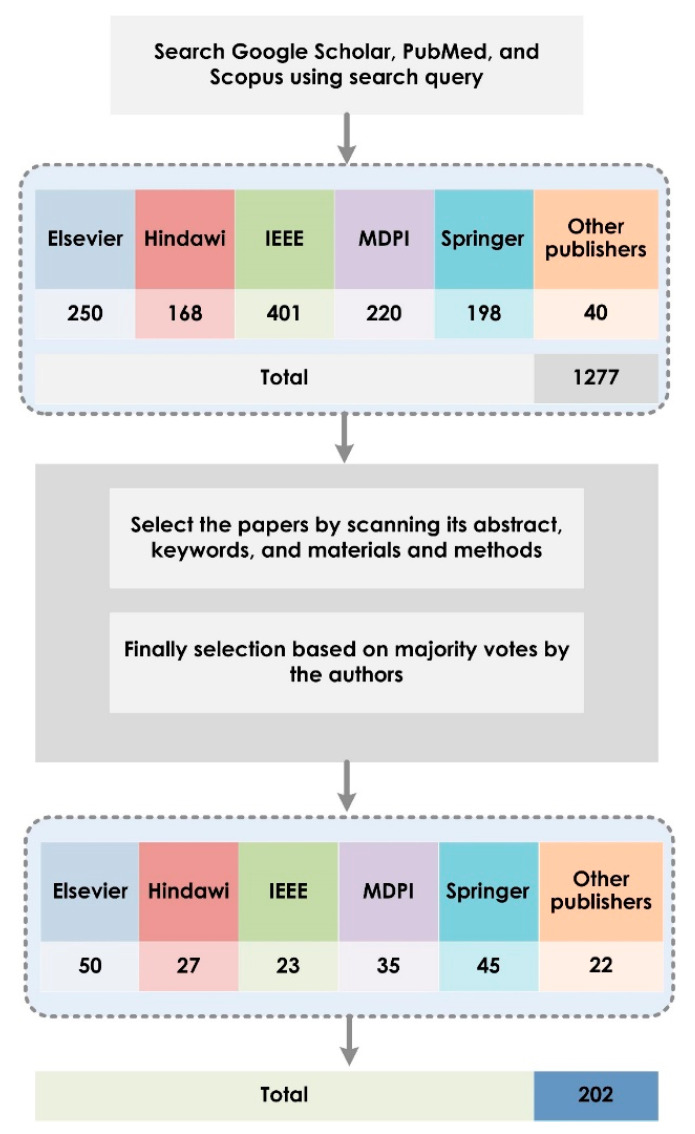
Overview of the selection process for relevant articles.

The relevance of a paper was based on title, abstract, materials and methods. An article was considered based on the voting scheme by the authors’ group of the current study. The authors are well-versed in the field of deep learning and machine learning techniques using various imaging modalities. Low-quality and conference papers were removed from the database. A final total of 202 papers (184 articles with 18 review papers) were compiled and analyzed. The selection process is shown in Figure 2. To the best of our knowledge, we have considered the data mining and deep learning research publications reported to present for identification of COVID-19 using various image modalities.

## 3. AI Techniques for COVID-19 Detection

**Figure 3:**
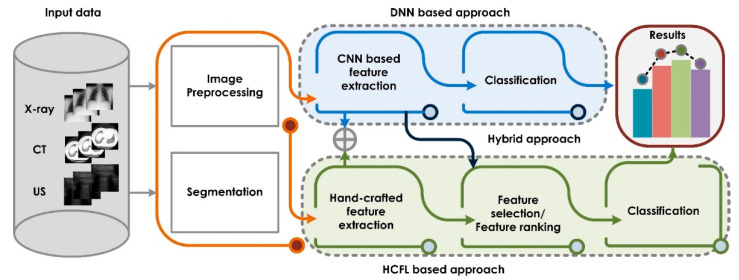
The complete framework to detect COVID-19 using various approaches.

Based on the state-of-the-art AI techniques to automatically detect COVID-19 using medical imagery, we categorized the methodologies as: (i) the DNN-based approach, (ii) the HCFL-based approach, and (iii) the hybrid approach. The input data consisted mainly of X-ray, CT, and US medical images of patients. In the DNN-based approach, convolutional neural networks (CNNs) are employed to automatically characterize the COVID-19 imagery. The DNN approach groups the feature extraction and classification components into an integrated neural network. In the HCFL-based approach, knowledge of features extraction techniques is required, followed by feature selection/ranking and classification stages. The hybrid approach fuses the methodologies from DNN- and HCFL-based approaches to obtain promising results. Figure 3 illustrates the key components used in the COVID-19 detection system.

### 3.1. COVID-19 Dataset: Medical Image

RT-PCR is the gold standard to diagnose COVID-19 using a nasal/throat swab. Sometimes the test results may not be available immediately and may cause a false negative result, due to the quality of the sample [31]. In such situations, various chest imaging modalities such as X-ray, CT, and Ultrasound (US) help to confirm COVID-19 suspects [32]. The combination of AI techniques with various imaging modalities can assist to increase the efficiency of COVID-19 detection worldwide [32].

**Figure 4:**
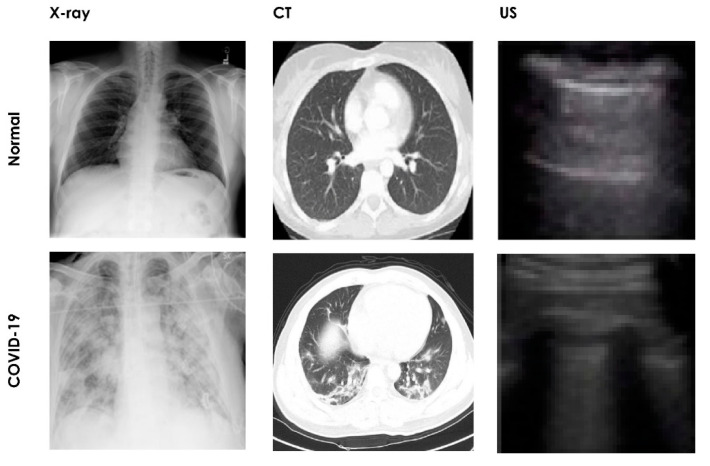
Sample images using various medical image modalities.

The development of an automated COVID-19 detection system based on chest X-ray imagery requires labeled images of normal and COVID-19 cases so as to train the system to differentiate healthy persons from COVID-19 patients. To test the system with an independent test dataset and to enhance its efficacy, it is necessary for these datasets to be made available publicly. With large datasets, it is possible for researchers to cross verify existing AI models before installation in hospitals or testing centers. Hence, medical images such as chest X-ray, CT, and lung US images are essential for the development of an automated COVID-19 detection system. Many researchers have of their own volition or in collaboration with hospitals, aggregated the COVID-19 datasets with various imaging modalities and released them publicly to assist research communities. Figure 4 shows examples of several chest images from publicly available datasets.

The majority of the state-of-the-art AI techniques depend on publicly available datasets (refer to Table 1). The first dataset uses the X-ray as the imaging modality, and is very popular due to the huge dataset collected from nine different sources and made available in a single source (refer to the given source in Table 1). It is noted that there are only a few public sources available for US images, compared to X-ray and CT images. In addition to the public datasets mentioned in Table 1, there are also other sources which have not yet been as widely utilized as an X-ray image source (available in: https://public.roboflow.ai/classification/covid-19-and-pneumoniascans (accessed on 19 July 2021)), for CT images [33], https://www.kaggle.com/andrewmvd/covid19-ct-scans (accessed on 19 July 2021), and for US images [34], https://github.com/jannisborn/covid19_ultrasound (accessed on 19 July 2021).

The X-ray images collected from various researchers in different parts of the world are available in portable network graphics format with the size of 299 × 299 pixels (https://www.kaggle.com/tawsifurrahman/covid19-radiography-database (accessed on 21 August 2021)). In [35], COVID-19 CT images were collected in various sizes from medRxiv (https://www.medrxiv.org/, latest accessed on 29 November 2021) and bioRxiv (https://www.biorxiv.org/, latest accessed on 29 November 2021), which was posted from 19 January to 25 March 2020. The minimum, average, and maximum widths are 124, 383, and 1485, respectively. The minimum, average, and maximum heights are 153, 491, and 1853, respectively [35]. In [36], CT scans have been collected from real patients in Sao Paulo Hospital, Brazil. It is also observed that the CT images were collected from municipal hospitals in Moscow, Russia. These are segregated based on severity i.e., CT1–CT4: COVID-19-related findings. The number of cases for each category is: CT0—254; CT1—684; CT2—125; CT3—45; and CT4—2 [37]. The largest publicly available lung US dataset was released in [39]. In total, 261 recordings (202 videos and 59 images) were gathered from 216 patients using either convex or linear probes. In addition, the British Society of Thoracic Imaging has also released a COVID-19 image database for teaching purposes (available in: https://www.bsti.org.uk/training-and-education/covid-19-bsti-imaging-database/ (accessed 19 July 2021)). Authors can use these underutilized datasets to enhance the heterogeneous capability of their own dataset. In addition, using the freely available datasets, researchers can initiate a community-oriented research effort to develop various models using AI techniques. Hence, it is also possible for the researchers to generalize their system using the various medical images.

### 3.2. Methodology

This section discusses the key processing stages covered by the different authors in the development of state-of-the-art COVID-19 detection systems.

#### 3.2.1. Preprocessing/Segmentation

Preprocessing is the initial stage used to enhance image quality by improving contrast and standardizing image pixel intensity levels. This stage plays a major role in obtaining accurate results. Usually, image quality is greatly improved by employing the contrast limited adaptive histogram equalization (CLAHE) technique [40]. Denoising techniques such as the Kirsch filter [41], Weiner filter [42], and pixel intensity normalization are also implemented. Other preprocessing techniques such as edge detection using the Prewitt filter (PF) [42], histogram equalization (HE), and gamma correction (GC) [43] may be useful. The aforementioned techniques are used in several works and can significantly increase the accuracy of the results.

For the CNN-based method, a common set of preprocessing techniques are employed. These techniques include resizing and shuffling. Furthermore, images are converted to RGB and then input to a CNN. In order to visualize the image more distinctly, the image boundaries are smoothed by normalization using morphological filters and by applying different filters and enhancement techniques. In addition, lung imagery is extracted using segmentation techniques such as region growing [44] and watershed [45], UNet [46], and LinkNet [47], where the latter is a variant of UNet and the variational data imputation method (VDI) [48].

In the process of training a deep learning model, sometimes there may be a shortage of datasets. In such situations, data augmentation techniques may be used to create additional data by slightly altering the existing data, thereby creating different versions of the original data. This acts as a regularizer and reduces overfitting while training the model. Data augmentation techniques such as rotation, cropping, flipping, and translation [49], Gaussian blur, and contrast adjustment have been used [50]. For the class imbalance, SMOTE [51] has been employed by several authors. Synthesis images can also be created using an adversarial network (GAN) [52], conditional GAN [53], auxiliary classifier generative adversarial network (ACGAN) [54] and Keras’ ImageDataGenerator (https://keras.io/api/preprocessing/image/ (accessed on 16 September 2021)).

#### 3.2.2. Feature Extraction

Feature extraction is mainly adapted to identify nonlinearities in the lung, thereby identifying lung abnormalities, if any. Several feature extraction techniques have been activated to detect COVID-19 more accurately. Handcrafted feature extraction methods such as the discrete wavelet transform (DWT) [55] and gray-level co-occurrence matrix (GLCM), and Haralick texture features [56] are the more commonly used methods. In addition, the features are also extracted with the two-dimensional (2D) curvelet transform (CTf) [57], residual exemplar local binary pattern (ResExLBP) [58], first order statistical features (FOSF) [50], histogram of oriented gradients (HOG) [59], dual-tree complex contourlet transform (DTCT) [60], local directional number pattern (LDN) [61], Pillow library [62] and fractional multichannel exponent moments (FrMEMs) [63], local binary pattern (LBP) [64], and multichannel fractional order Legendre Fourier moments (MFrLFM) [65], to characterize textural information.

Similarly, features models have also been extracted using a CNN-based approach. In this approach, base architectures such as ResNet101 [66], AlexNet [67], DenseNet-201 [68], VGG16 [69], GoogLeNet [70], MobileNetv2 [71], Inceptionv3 [72], SqueezeNet [73], VGG19 [74], and Xception [75] have been adjusted for feature learning and extraction. Transfer learning (TL) has been arrayed to cope with the limitations that arise from lack of freely accessible labeled medical images. In addition to TL, methods such as the multilayer perceptron convolutional neural network (MLP-CNN) have been assembled to handle mixed data types consisting of numerical/categorical and image data [76]. Similarly, a high-resolution network (HRNet) has been used for extracting detailed features [77]. In addition, the authors have also furnished customized CNN models to improve system performance.

#### 3.2.3. Feature Selection/Optimization

Feature selection is employed to reduce redundant content by preserving significant information. The sequential feature selector algorithm (SFS) [78], chaotic salp swarm algorithm (CSSA) [79], advanced squirrel search optimization algorithm (ASSOA) [80], and harmony search (HS) [81] algorithm are extensively utilized to reduce redundant information in feature representation. Similarly, ReliefF and Neighborhood Component Analysis (NCA) are used to select optimal features, i.e., RFINCA [82]. In addition, methods such as binary gray wolf optimization (GWO) [83] and hybrid social group optimization (HSGO) [84] have proven their efficacy in providing best optimized features. Scientists have also fitted the fractional-order marine predators algorithm (FO-MPA) [85], minimum redundancy and maximum relevance (mRMR) [86], and manta ray foraging optimization (MRFO) [63] in order to select the most significant features. Feature dimensionality reduction has been undertaken using a t-distributed stochastic neighbor embedding (t-SNE) technique [87] and principal component analysis (PCA) [88]. Apart from these methods, feature selection using mutual information (MI) [89], Relief-F [90] and the dragonfly algorithm (DA) [91], and the guided whale optimization algorithm (Guided WOA) [92] have also been set up. In addition, feature selection has been performed using maximum entropy and ANOVA test [93].

Because optimizers are the crucial part of the neural network, the most commonly used algorithms for DNN approaches are the stochastic gradient descent, adaptive learning rate optimization algorithm [94], and root mean square propagation [95], which are supplied to update the network weights. CNN with GWO and whale optimization with the BAT algorithm have been employed to tune the hyperparameters [96,97]. Furthermore, biogeography-based optimization [98], and the multi-objective differential evolution (MODE) parameter tuning method have been used to optimize the parameters [99].

#### 3.2.4. Classification

In the classification stage, a decision is made on test images by predicting the labels. In order to categorize COVID-19 infections, highly accurate classifier techniques play an important role. Classifier techniques such as random forest (RF) [100], the support vector machine (SVM) [101], and the bagging tree classifier [102] have proven their efficacy in multiclass classifications. In addition to these classification techniques, k-nearest neighbor (k-NN) [103], decision tree (DT) [104], Naïve Bayes (NB) [105] and artificial neural network (ANN) [106], generalized regression neural network (GRNN) [107], MLP neural network [108], probabilistic neural network (PNN) [109], and extreme learning machine (ELM) [110] classifier are also used by the research community. Moreover, adaptive boosting (AdaBoost) [111], eXtreme Gradient Boosting (XGBoost) [112], and logistic regression (LR) [113] have also been incorporated by various investigators. However, the authors selected the classifiers based on the best achieved results for the extracted features. Table 2, Table 3, Table 4 and Table 5 are summaries of state-of-the-art techniques used in the automated detection of COVID-19 with various image modalities.

## 4. Results

From our extensive literature review, it was observed that many of the CAD tools in the area of several medical fields have used accuracy, sensitivity or recall, specificity, positive predictive value (PPV) or precision, F-measure or F-score, and area under the curve (AUC) to evaluate the performance of the system [274,275,276]. Similarly, the performance of the CAD tool for the identification of COVID-19 was also evaluated using the same performance parameters as mentioned above. Let TP, TN, FP, and FN indicate true positive, true negative, false positive and false negative, respectively. They are given by the following equations:

Accuracy = (TP + TN)⁄(TP + TN + FP + FN)(1)

Sensitivity = TP⁄(TP + FN)(2)

Specificity = TN⁄(TN + FP)(3)

PPV = TP⁄(TP + FP)(4)

F1-score = 2TP/(2TP + FP + FN)(5)

**Figure 5:**
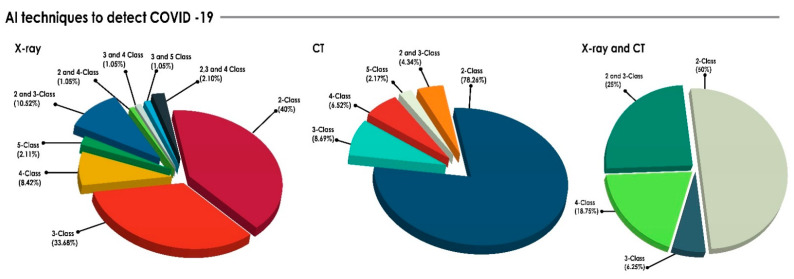
Percentage of various classes in the assessment of COVID-19 by imaging modalities (X-ray, CT, and X-ray and CT).

In all performance measures, the higher the value, the better the performance of the model. The developed AI models for COVID-19 detection using various medical images, such as X-ray, CT, and US, can be categorized into 2, 3, 4, and 5 classes per imaging modality, as shown in Figure 5.

Figure 5 reveals that two-class classification (Healthy vs. COVID-19 or COVID-19 vs. NonCOVID-19) was the most frequently reported among the different imaging modalities. Combinations of different class categorizations were also observed in CADTs which used X-ray images. Table 6 conveys the average performance outcomes of the systems considered in the present review irrespective of the number of cases. Many of the studies used publicly available datasets and achieved comparable results.

**Figure 6:**
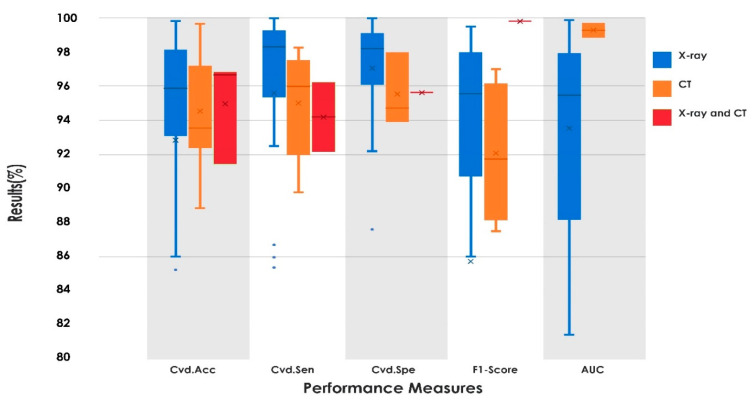
Comparison of Cvd.Acc, Cvd.Sen, Cvd.Spe, F1-Score, and AUC of AI techniques to detect COVID-19 using box plots.

It is observed from Table 6 that the systems developed with X-ray and CT images had five-class classification and achieved a Cvd.Acc (avg.) of 92.41% using X-ray images. It is also observed that the two-class models are no longer valid when other diseases with similar symptoms were presented [178]. It is noted from Table 2, Table 3, Table 4 and Table 5 that few studies have performed four-class (normal vs. COVID-19 vs. viral pneumonia (VP) vs. bacterial pneumonia (BP)) classification [114,118,138,154,161,179,189,194,264]. They have obtained the Cvd.Acc (avg.) of 89.91%. Hence, for further analysis of the system we considered the model which can categorize three or more images. Box plot analysis was carried out to obtain the overall performance of the three-class classification system used in COVID-19 detection. Figure 6 shows the box plots for Cvd.Acc, Cvd.Sen, Cvd.Spe, F1-Score, and AUC values of the reported AI methods in the three-class classification scenario. Box plots represent the distribution characteristics of performance measures based on minimum, first quartile, median, third quartile, and maximum.

It is noted from Figure 6 that AI techniques using X-ray imagery had acceptable performance when compared to other medical images. For the three-class scenario, the method achieved Cvd.Acc (avg.) of 94.78%, 94.55%, and 94.99% using X-ray, CT, and the system with both X-ray and CT, respectively, by considering all state-of-the-art techniques. Further, we also analyzed the systems which can categorize three or more classes. It is observed from Table 2 that ResNet50 with DWT and GLCM [114], customized CNN [118,154,179,189], GoogLeNet [138], InceptionNet [141], AlexNet [160], a combination of DenseNet103 and ResNet18 [148], an ensemble of various models such as InceptionResNetV2, ResNet152V2, VGG16, and DenseNet201 [153], and a grouping of MobileNet and InceptionV3 [161] were effectively used for four-class classification using X-ray images. Further, the authors also used CNN models for five-class classification using X-ray images [129,168,177]. From Table 2, it is also noted that only RF [114], SVM [179], and ensemble of classifiers [194] have achieved comparable results for four-class categorization. Herein, the RF classifier shows its suitability multiclass categorization by achieving Cvd.Acc of 98.48%. From Table 3, it is observed that grouping of ResNet152V2, DenseNet201, and VGG16 [212], deep learning model [216], and PSSPNN [232] were used by the authors to categorize four-class CT images. The combination of various DNN models achieved a Cvd.Acc of 98.83% [212]. From Table 4 it is noted that minimal work has been reported using lung US imagery. In [258] the autoencoder and modified DenseNet201 is used for four-class classification, and achieved a better result, by over 17%, compared to traditional DenseNet. In [260,264], the system is tested with X-ray and CT modalities, and achieved better classification for four classes. The usage of VGG19 [260] and VGG16 [264] have shown their significance in four-class classification, as noted in Table 5. In [265], a combination of DenseNet103 with Haralick textural features and the ResNet101 model also showed promising performance. It is furthermore observed that for all modalities, only the VGG19 model is used for three-class categorization [273]. It achieved better result for US images, when compared to X-ray and CT.

## 5. Discussion

**Figure 7:**
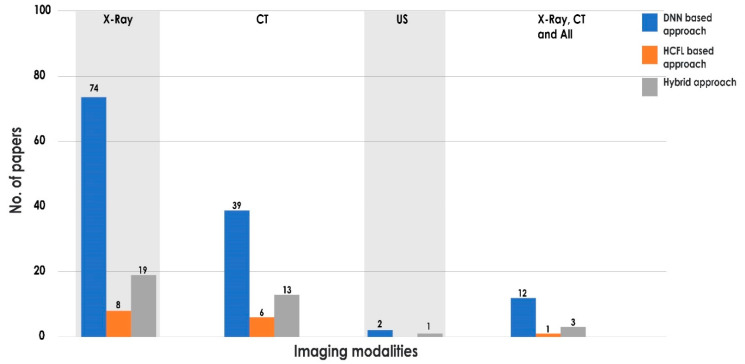
Various methodologies adopted by state-of-the-art techniques using different modalities.

Investigators have developed many models to detect COVID-19 during the past two years and have shown that there is a role for AI in detecting COVID-19 [19,21,22,23,24,25,26,27,28,29,277,278,279,280,281]. The 184 technical papers reviewed in this study provide up-to-date knowledge on the usage of AI techniques in detecting COVID-19. The developed models were categorized based on DNN, HCFL, and hybrid methodologies. The number of articles based on the three methodologies are highlighted in Figure 7.

It is observed from Figure 7 that 70% of the papers reported the use of a DNN-based approach, which included pre-trained networks and customized CNNs. Very few papers were developed to quantify the severity of COVID-19 [282,283,284,285,286]. It is also noted that the computational cost of various deep learning approaches is high [287,288]. From Figure 5, 40%, 78.26%, and 50% of the papers using X-ray, CT, and all modalities, respectively, reported only two-class classification. However, it is difficult to show its significance level in real-time to categorize multiple classes with similar symptoms. It is also observed from Table 6 that, for four-class classification, the Cvd.Sen and Cvd.Spe of the methods increased 4.5% and 1.66%, respectively, using CT images, when compared to X-ray images. In most of the cases, CNNs were able to successfully extract significant information from lung tissue with pneumonia, (i.e., BP and VP). Pre-trained networks such as ResNet, DenseNet, and VGG were successfully used in all of the modalities for greater than three-class categorization. However, the comparison of the pre-trained networks for binary classification may not be as useful, since it may fail to distinguish diseases which have similar symptoms with COVID-19.

In short, it is very difficult to make a comprehensive comparison of methodologies in this present situation because the methods were evaluated using various datasets of different sizes. Hence, the general opinion on the algorithm may be reduced. Few investigators performed k-fold cross validation and in most of the cases the hold-out method was used. Therefore, it is difficult to observe the consistency in the developed models.

Although several models have been developed to detect COVID-19, there are many factors involved in the analysis of COVID-19 imagery, which are listed as follows:

***Implementation of multiclass categorization models***: Many of the studies implemented two-class categorization; however, these are restricted to only understanding the features of normal and COVID-19 images. For disease symptoms similar to COVID-19, there is a need for algorithms which can discriminate among various classes, such as normal, COVID-19, pneumonia, BP, VP, tuberculosis, and lung opacity. Hence, there is a need for models which can understand the inherent characteristics of various diseases and predict the severity level. Investigators should therefore concentrate on the generalization aspects of the developed models by considering all image modalities.

***Implementation aspects***: State-of-the-art techniques have trained models using a transfer learning approach. Although the results are promising, the primary architecture has been developed to handle real-world color images. Hence, there is a need for DNNs which are trained from scratch using real medical images. In addition, the selection of appropriate hyper parameters to obtain improved accuracy will play a significant role in training networks developed in the future. The discrimination power of AI techniques can be improved by training the system with multiple views of medical images, which, however, requires extra time. Hence, there is a need for compact featuring to represent COVID-19 and other similar diseases to handle huge datasets.

***CADTs to analyze prognosis of COVID-19***: Researchers should exploit the hybrid methodology to help medical doctors to understand the treatment outcomes for COVID-19. It is important to develop models to assess the health condition of post-COVID-19 patients for better health and management of the system.

### 5.1. Future Trends

Since the onset of the COVID-19 pandemic, home isolation and quarantine have been implemented by governments across the world to control the spread of the pandemic [289,290]. In addition, risk factors such as fever, weakness, heart disease, and dry cough, are the most critical issues in the mortality of patients [291]. A person who has tested positive for COVID-19 or who has been in close contact with a confirmed COVID-19 person has to undergo a period of quarantine. In cases where home quarantine is required, especially in rural areas of developing countries, the hospital may require frequent health updates from the patient. This can be done via smartphone where the patient monitors his/her own temperature and/or SpO_2_ level and reports the results to the medical doctor. In this way the doctor is able to monitor patient health remotely and provide suitable prescriptions or medications when required. There is also a chance that the results obtained from the antigen rapid self-test kit may be negative, despite the patient showing symptoms of COVID-19 disease. In addition, there may be other issues such as people with disabilities and elderly people dependent on them. Considering all of these issues, the best solution would be to remotely monitor the patient without the need for frequent visits to the hospital.

**Figure 8:**
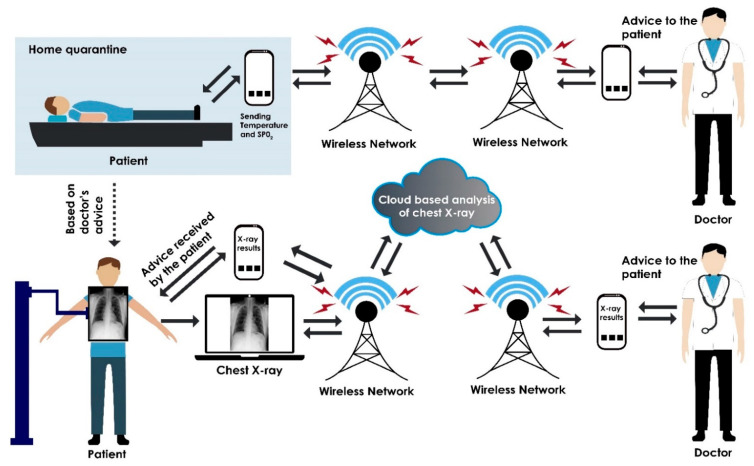
IoT-based smart healthcare system to detect COVID-19.

Recent advancements in the Internet of Things (IoT) have paved the way for providing improved healthcare support services [292]. In the future, a cloud-based wireless healthcare system can be used to control the observation of COVID-19 epidemiologically, as shown in Figure 8. X-ray images of the patient’s chest can be taken at selected rural hospitals. X-ray imaging is a fast, inexpensive, and minimally invasive procedure, and X-ray units are available in most rural hospitals. Before collecting the data, the institute’s ethical committee approval should be granted, and the imaging data should be collected after obtaining written consent from the patients. The collected data are stored in a secured cloud-based server with unique identification number for each patient. X-ray images are then analyzed using a cloud-based system, and observations are sent to the medical doctors. On close examination of the imagery, the doctor provides suitable advice to the patient along with prescriptions and treatment instructions. Hence, medical doctors and their patients can interact remotely for any further treatment even in rural communities.

### 5.2. Limitations of the Review

**1.** This review considered only manuscripts written in English.

**2.** In this review process, many databases were explored using different search queries; thus a few relevant works may have been neglected in the search. The review process was performed based on technical papers to detect COVID-19 rather than on clinical studies.

**3.** The present work provides a systematic review of AI techniques, analysis, and its advancement. However, the transformation before and after COVID-19 is not assigned great importance in this study.

The scope of this review was the comprehension of the AI techniques using different imaging modalities. It is observed that the CT scan, which is the faster and more feasible method, has been proven to be the most sensitive tool in the diagnosis of COVID-19 compared to the RT-PCR test [293]. However, the technique involves a high dose of radiation and is not available in the rural health care sectors in developing countries [294,295]. In contrast, the chest X-ray is a universally available technique with 30–70 times lower radiation exposure, and the test is performed during the initial investigational process for COVID-19 [296]. However, lung US is an alternative mode that produces results similar to those of the chest CT and is considered to be superior to the chest X-ray in the diagnosis of lung pathology in COVID-19 infection. Nonetheless, this modality is not useful when the pleura is spared from the pneumonic pathology during the early course of the disease [297]. Recent developments in the diagnosis of COVID-19 using signals such as respiratory sounds, speech signals, and coughing sounds, have also attracted many researchers [298,299]. Furthermore, in the future, this can be combined with other imaging modalities and signals to enhance the performance of the system using various deep learning approaches.

## 6. Conclusions

AI techniques do not substitute for medical doctors and expert radiologists. However, they can efficiently and automatically impact the analysis of medical imagery. The development of CAD tools to detect COVID-19 have grown significantly in recent years, contributing to the body of clinical and medical research. The early detection of COVID-19 using AI techniques would be helpful to prevent the progression of the pandemic by enabling rapid decision-making. This study aimed to observe and analyze the growth and improvement in AI techniques for the detection of COVID-19. In this review, 184 papers were selected and summarized. The results showed that all DNN, HCFL, and hybrid approaches have high a potential to predict COVID-19 cases. The classification, segmentation, and quantification of the severity level of COVID-19 on heterogeneous datasets can be improved if medical experts play a significant role in building the framework for AI techniques, providing significant knowledge of image features and real-world requirements.

## Figures and Tables

**Table 1:** Summary of frequently used publicly available datasets for the detection of COVID-19.

S.No.	Paper/Source	Imaging Modality	Total Number of Images
1	Available in: https://www.kaggle.com/tawsifurrahman/covid19-radiography-database (accessed on 21 August 2021)	X-ray	Normal: 10,192 COVID: 3616 Viral Pneumonia:1345 Lung opacity: 6012
2	Available in: https://www.kaggle.com/prashant268/chest-xray-covid19-pneumonia (accessed on 21 August 2021)	X-ray	Normal: 1583 COVID: 576 Pneumonia: 4273
3	[35]/Available in: https://github.com/UCSD-AI4H/COVID-CT (accessed on 21 August 2021)	CT	COVID:349 NonCovid: 397
4	[36] Available in: https://www.kaggle.com/plameneduardo/sarscov2-ctscan-dataset (accessed on 21 August 2021)	CT	COVID:1252 Noncovid:1230
5	[37]/Available in: https://mosmed.ai/datasets/covid19_1110 (accessed on 21 August 2021)	CT	1110 patients with severity grading (CT-0 to CT-4)
6	[38]/Available in: https://zenodo.org/record/3757476#.YPUTnugzbIU (accessed on 21 August 2021)	CT	20 labeled COVID-19 CT scans (1800 + annotated slices)
7	[39]/Available in: https://github.com/BorgwardtLab/covid19_ultrasound (accessed on 21 August 2021)	US	Videos and images Healthy: 90 COVID-19: 92 Bacterial Pneumonia: 73 Viral Pneumonia: 6

**Table 2:** State-of-the-art AI techniques to detect COVID-19 using chest X-ray imagery.

Paper	Method Used: Preprocessing + Segmentation + Feature Extraction + Feature Selection + Classification or CNN + Classification	Result Obtained	Dataset Used (Most Are Public)		No. of Classes
[114]	Image enhancement + WS +deep CNN (ResNet50) and DWT and GLCM+ mRMR+ RF	Cvd.Acc: 99.45, Cvd.Sen:.99.17, Cvd.Pre: 97.51,F1-Score: 0.9833	N:1500,C-19: 790,BP: 1304,VP: 1215 (after data augmentation)		2 (C-19, NC)
		Cvd.Acc: 98.48, Cvd.Sen: 98.72, Cvd.Pre: 97.89,F1-Score: 0.9829			4
[115]	Color layout descriptor + *k*-NN	Cvd.Sen: 96.5, Cvd.Pre: 96.5	Total:86		
[116]	CNN model + Long short-term memory (LSTM)	Cvd.Acc: 99.4, Cvd.Sen: 99.3, Cvd.Spe: 99.2, F1-Score: 98.9, AUC: 99.9	N: 1525, C-19: 1525,P: 1525		3
[117]	Concatenation of the Xception and ResNet50V2	Cvd.Acc (avg.): 91.4	N: 8851,C-19: 180,P: 6054		3
[118]	CNN model	Cvd.Acc: 95, Cvd.Sen: 96.9, Cvd.Spe: 97.5, Cvd.Pre: 95, F-measure: 95.6	N: 310,C-19: 284,BP: 330,VP: 327		3(N, C-19, P)
		Cvd.Acc: 89.6, Cvd.Sen: 89.92, Cvd.Spe: 96.4, Cvd.Pre: 90,F-measure: 96.4			4
[119]	CNN model	AUROC: 0.96	Pvt. + Public Dataset		3
[120]	DarkNet based CNN model	Cvd.Acc(avg.): 98.08, Cvd.Sen(avg.): 95.13, Cvd.Spe(avg.): 95.3, Cvd.Pre (avg.): 98.03,F1-Score (avg.): 96.51	N: 500,C-19: 127,P: 500		2 (N, C-19)
		Cvd.Acc(avg.): 87.02, Cvd.Sen(avg.): 85.35, Cvd.Spe(avg.): 92.18, Cvd.Pre (avg.): 89.96,F1-Score (avg.): 87.37			3
[121]	2D-CTf + CSSA+ EfficientNet-B0	Cvd.Acc: 99.69, Cvd.Sen: 99.44, Cvd.Spe: 99.81, Cvd.Pre: 99.62, F-measure: 99.53	N: 1281,C-19: 159,VP: 1285		3
[122]	VGG-16 model	Cvd.Acc(avg.): 97	N: 3520,C-19: 250,P: 2753		3
[123]	ResNet50 + ResNet101	Cvd.Acc: 97.77, Cvd.Sen: 97.14, Cvd.Pre: 97.14	N: 315,C-19: 250, BP: 300,VP: 350		2(C-19,O)
[58]	ResExLBP + Relief-F+ SVM	Cvd.Acc: 99.69, Cvd.Sen: 98.85, Cvd.Spe: 100	N: 234, C-19: 87		2
[124]	VGG16 model	Cvd.Acc: 98.1	N: 2880, C-19: 415, P: 5179		2(C-19,NC)
		Cvd.Acc: 94.5			3
[125]	ResNet18, ResNet50, SqueezeNet,& DenseNet121	Cvd.Sen: 98, Cvd.Spe(avg.): 90	C-19: 200, NC:5000		2
[126]	Capsule Network-based architecture	Cvd.Acc: 95.7, Cvd.Sen: 90, Cvd.Spe: 95.8, AUC: 0.97			2(C-19,O)
[127]	VGG16 model	Cvd.Sen: 97.62, Cvd.Spe: 78.57	N:142, C-19: 142		2
[128]	ResNet101	Cvd.Acc: 71.9, Cvd.Sen: 77.3, Cvd.Spe: 71.8	C-19: 154, NC: 5828 (test data)		2
[129]	Deep learning model	Cvd. Acc C-19: 100,P: 93.75,N: 100	N: 66, C-19: 51,NC: 21,P: 160,TB: 54		5
[130]	Sequential CNN model	Cvd.Acc: 98.3, Cvd.Sen: 100, Cvd.Pre: 96.72, F1-Score: 98.3,ROC area: 0.983	N: 659, C-19: 295		2
[131]	HE +VGG16-based model	Cvd.Acc (avg.): 86, Cvd.Sen (avg.): 86, Cvd.Spe(avg.): 93, Cvd.Pre(avg.):86,F1-Score: 86	N: 132, C-19: 132,P: 132		3
[132]	Histogram matching and autoencoder and CLAHE + Custom CNN model	Cvd.Acc (avg.):94.43, Cvd.Sen (avg.): 92.53, Cvd.Spe: 96.33, Cvd.Pre(avg.): 93.76, F1-Score (avg.): 93.14,AUC (avg): 0.988	N: 4337,C-19: 2589		2
[133]	Ensemble of ResNet-18 Model	Cvd.Acc: 95.5, Cvd.Sen: 100, Cvd.Pre: 94	N: 1579,C-19: 184,P: 4245		3
[134]	HE+ lung segmentation using UNet + Various deep model are analyzed.				
[135]	4 models analyzed (Best: VGG16 and VGG19)	Cvd.Acc: 99.38, Cvd.Sen: 100, Cvd.Spe: 99.33	N: 802, C-19: 790		2
[136]	CLAHE+VGG16 and VGG19 used (Best: VGG16)	Cvd.Acc: 95.9, Cvd.Sen: 92.5, Cvd.Spe: 97.5,AUC: 0.950 (max. only for C-19)	N: 607,C-19: 607,P: 607		3
[137]	CNN model to separate COVID-19 and pneumonia				
[138]	Alexnet, Googlenet, and Restnet18 is used (Googlenet best for 4 classes)	Cvd.Acc: 80.56, Cvd.Sen: 80.56, Cvd.Pre: 84.17, F1-Score: 82.32	N: 79,C-19: 69, BP: 79, VP: 79		4
[76]	MLP-CNN	Cvd.Acc: 95.4, Cvd.Sen: 95, Cvd.Pre: 92.5, F1-Score: 93.6	C-19: 112, NC: 30		2
[139]	LightCovidNet	Cvd.Acc (avg.): 96.97	N: 1341,C-19: 446,P: 1345		3
[140]	MobileNet v2	Cvd.Acc: 96.78, Cvd.Sen: 98.66, Cvd.Spe: 96.46	N: 504, C-19: 224, P: 714		2(C-19,O)
		Cvd.Acc: 94.72			3(N,C-19,P)
[141]	Truncated InceptionNet	Cvd.Acc (avg.): 98.77, Cvd.Sen(avg.): 95, Cvd.Spe(avg.): 99, Cvd. Pre(avg.): 99 F1 score(avg.): 0.97, AUC (avg.):0.99	N:2003, C-19:162,P: 4280, TB:400		4
[142]	CNN model	Cvd. Prec (avg.), Cvd. Sen (avg.), F1-score (avg.): 100	C-19: 500, P: 500		2
[143]	CNN model	Cvd.Acc (testing): 94.4	N:8066, C-19:183,P: 5551		3
[144]	COVID-Net model	Cvd.Acc: 93.3	Total: 13,975 from 13,870 patients		3(N,C-19,P)
[85]	CNN model (Inception) + FO-MPA + *k*-NN	Cvd.Acc: 98.7, F-score: 98.2	DS1: C-19 +ve: 200, C-19 -ve: 1675		2
		Cvd.Acc: 99.6, F-score: 99	DS2: C-19 +ve: 219, C-19 -ve: 1341		
[63]	FrMEMs + MRFO + *k*-NN	Cvd.Acc: 96.09, Cvd.Sen: 98.75, Cvd.Pre: 98.75	DS1: C-19 +ve: 216,C-19 -ve: 1675		2
		Cvd.Acc: 98.09, Cvd.Sen: 98.91, Cvd.Pre: 98.91	DS2: C-19 +ve: 219,C-19 -ve: 1341		
[145]	Xception model + SVM	Cvd.Acc: 99.33, Cvd.Sen: 99.27, Cvd.Spe: 99.38, Cvd.Pre: 99.27, F1-score:99.27,AUC: 99.32	N: 565,C-19: 537		2
[146]	Discriminative cost sensitive learning approach	Cvd.Acc: 97.01, Cvd.Pre: 97, Cvd.Sen: 97.09,F1-score: 96.98	N: 1000,C-19: 239,P: 1000		3
[147]	CNN model	Cvd.Sen (avg.): 91.05, Cvd.Spe(avg.): 99.61, Cvd.Acc(avg.): 98.34,ROC-AUC(avg.): 95.33	N: 1583,C-19: 225		2
		Cvd.Sen (avg.): 92.88, Cvd.Spe(avg.): 99.79, Cvd.Acc(avg.): 99.44,ROC-AUC(avg.): 96.33	C-19: 225, P: 4292		2
		F1 score (avg.): 94.10	N: 1583,C-19: 225,P: 4292		3
[148]	HE and GC + DenseNet103 + ResNet18	Cvd.Acc: 91.9	N: 191, C-19: 180,BP: 54, VP: 20,TB: 57		4(N,BP,VP,TB)
[149]	VGG16 model	Cvd.Acc, Cvd.Sen, Cvd. Prec, F-score: 80	C-19: 70, NC: 70		2
[54]	ACGAN based model (CovidGAN)	Cvd.Acc: 95.00	N: 403, C-19: 721		2(N, C-19)
[150]	CNN model	Cvd.Acc: 99.70, Cvd.Pre: 99.70, Cvd.Sen: 99.70, Cvd.Spe: 99.55	N: 1579, C-19: 423,VP:1485		2(N,C-19VP)
[151]	Deep learning model	Cvd.Acc: 97.25, Cvd.Pre: 97.24,F1-score: 97.21	N: 27,228, C-19: 209, P: 5794		3
[152]	CNN + gated recurrent unit (GRU)	Cvd.Sen: 96, Cvd.Pre: 96, F1-score: 95	N: 141, C-19: 142, P: 141		3
[153]	Ensemble of deep CNN model (InceptionResNetV2 + ResNet152V2 + VGG16+ DenseNet201)	Cvd.Acc: 99.2, Cvd.Sen: 99.12, Cvd.Spe: 99.07, F-score: 99.17,AUC: 99.21	N:2039, C-19:1663,P: 401,TB:394		4
[154]	MCFF-Net66-Conv1-GAP	Cvd.Acc: 94.66	N:1500,C-19:942, BP:1802,VP:1797		4
[155]	ResNet50V2 + t-SNE	Cvd.Acc: 95.49, Cvd.Sen: 99.19, Cvd.Pre:96.19, F1-score: 98.0, AUC: 95.49	N: 616, C-19: 616,P: 616		3
[156]	CNN model	Cvd.Acc:100, Cvd.Sen:100, Cvd.Spe:100, Cvd.Prec:100, F1-score:100, AUC:100	N:42, C-19:136		2
[157]	Enhanced Inception-ResNetV2 model	Cvd.Acc(avg.): 98.80, Cvd.Sen(avg.): 99.11, Cvd.Prec(avg.): 98.61,F1 score(avg.): 98.86	N:1341,C-19:219,VP: 1345		3
[158]	CNN model and GoogLeNet	Cvd.Acc: 97.62, Cvd.Sen: 98.29, Cvd.Spe: 97.64, F-score: 98.30,AUC: 97.96	N: 1421,C-19: 1332		2
[159]	VGG16 Model	Cvd.Acc: 98.72, Cvd.Sen: 98.78, Cvd.Spe: 98.70, Cvd.Prec: 96.43, F1-score: 97.59	N:1341,C-19:1200,VP:1345		3
[160]	AlexNet	Cvd.Acc: 99.13, Cvd.Sen: 99.4, Cvd.Spe: 99.15,F-score: 99.49,AUC: 99.31	Consists: N,C-19,P,TB		4
[161]	Ensemble of MobileNet and InceptionV3	Cvd.Acc: 96.49, Cvd.Prec: 93.01, Cvd.Sen: 92.97,F-score: 92.97	N:1050,C-19:1050,BP:1050,VP:1050		4
[162]	VGG16 model	Cvd.Acc(avg.): 91.69, Cvd.Sen(avg): 95.92, Cvd.Spe(avg.): 100	Total: 7720		3(N, C-19,P)
[163]	CLAHE + InceptionV3 + ANN	Cvd.Acc: 97.19	N: 1583,P: 4273		2
[97]	CNN with various optimization algorithm	Cvd.Acc:96, Cvd.Sen:100, Cvd.Spe:99, Cvd.Pre:96, F1-Score:0.98	N: 1583, C-19: 576, VP:4273		3
[164]	VGG16 model	Cvd.Acc: 96, Cvd.Sen: 92.64, Cvd.Spe: 97.27	N: 504, C-19: 224		2
		Cvd.Acc: 92.53, Cvd.Sen: 86.7, Cvd.Spe: 95.1	N:504, C-19: 224, P: 700		3
[50]	FOSF and GLCM and HOG + GWO + Ensemble of classifiers	Cvd.Acc: 98.06, Cvd.Sen: 98.83, Cvd.Spe: 96.51, Cvd.Pre: 98.26,F-measure: 98.55 AUC:0.97	N: 782, C-19: 782, P: 782		2 (N,AB)
		Cvd.Acc: 91.32, Cvd.Sen: 96.51, Cvd.Spe: 86.2, Cvd.Pre:87.36,F-measure: 91.71,AUC: 0.91			2(C-19,P)
[165]	Ensemble of deep CNN model (VGG19 + DenseNet121) + SVM	Cvd.Acc: 99.71	N:2341, C-19: 798,P: 2345		2 (C-19,NC)
		Cvd.Acc: 98.28, Cvd.Sen (avg), Cvd.Pre(avg.),F1-Score (avg.): 98.33			3
[166]	CNN model + Ensemble of classifiers	Cvd.Acc: 98.91, Cvd.Sen: 97.82, Cvd.Pre: 100,F1-Score: 98.89	N: 2300,C-19: 2300		2
[167]	Deep learning model (Inception architecture)	Cvd.Acc: 96, Cvd.Sen: 93, Cvd.Spe: 97, Cvd.Pre: 97, F1-Score: 0.96	C-19: 435,NC: 505		2
[168]	UNet with ResNet + CNN model	Cvd.Acc (avg.): 96.32	N:1840,C-19:433,BP:2780,VP:1345,TB: 394		5
[169]	Two separate CNN models for binary and ternary classification	Cvd.Acc: 98.7, Cvd.Sen: 100, Cvd.Spe: 98.3	N:145,C-19: 145, BP: 145		2(N, C-19)
		Cvd.Acc: 98.3, Cvd.Sen: 99.3, Cvd.Spe: 98.1			3
[170]	VGG16 and Xception model (Best: Xception)	Cvd.Sen: 100, Cvd.Spe: 97.6, F1-Score: 97.7	N: 400, C-19: 402,P:200,I: 35		2
[171]	Various DNN + Majority voting scheme	Cvd.Acc: 99.31	N: 1338, C-19: 237, VP: 1336		3
[172]	Customized CNN Model	Cvd.Acc: 92.95, Cvd.Sen (avg.): 90.72, Cvd.Pre(avg.): 94.04,F1-Score(avg.): 0.9204	N: 1341, C-19: 744 (Independent set)		2
[173]	NanoChest-net model	Analyzed with various datasets.			
[174]	VGG16+ HS + *k*-NN	Cvd.Acc, Cvd.Sen, Cvd.Pre,F1-Score, AUC:100	N: 480,C-19: 280		2
[175]	OptiDCNN model	Cvd.Acc: 99.11	N: 5000, C-19: 184		2
[176]	HOG and CNN(VGG19) + ME + CNN classifier + WS	Cvd.Acc: 99.49, Cvd.Sen: 93.65, Cvd.Spe: 95.7	C-19 +ve: 1979, C-19 -ve: 3111		2
[177]	Ensemble-CNNs (based on ResNeXt-50, Inception-v3, and DenseNet-161)	Cvd.Acc: 75.23 ± 3.40, Cvd.Sen: 75.20, Cvd.Spe: 87.60, Cvd.Pre: 78.28, F1-Score: 73.43 AUC: 0.8140	N: 711, C-19: 711,P:711,BP:711,VP:711 Lung Opacity not Pneumonia:711 (public+Pvt.)		3(N,C-19,P)
		Cvd.Acc: 81.00 ± 2.39, Cvd.Sen: 82.96, Cvd.Spe: 85.24, Cvd.Pre: 82.99,F1-Score: 81.49, AUC: 0.8810			5
[178]	Showed that a system with 2-class model are not valid for the diseases with similar symptoms, by conducting various experiments				
[179]	Exemplar COVID-19FclNet9 + SVM	Cvd.Acc: 99.64	N: 150,C-19:127		2
		Cvd.Acc: 98.84	N: 4000,C-19: 3616, P: 1345		3
		Cvd.Acc: 97.60	N: 234,C-19:125,BP:242,VP:148		4
[180]	Decompose, Transfer, and Compose (*DeTraC*)+PCA	Cvd.Acc: 93.1, Cvd.Sen:100	N: 80, C-19:105,SARS: 11		3
[77]	UNet + HRNet	Cvd.Acc: 99.26, Cvd.Sen:98.53, Cvd.Spe: 98.82	Total: 272		2
[181]	Various CNN model used (Best:EfficientNetB0)	Cvd.Acc:92.93, Cvd.Sen: 90, Cvd.Spe: 95, Cvd. Prec: 88.3,F1- score: 0.88	N: 1341, C-19: 420, P: 1345		3
[182]	EfficientNet B3-X	Cvd.Acc: 93.9, Cvd.Sen: 96.8, Cvd.PPV: 100	N:7966+100, C-19: 152+31 P: 5421+100		3
[183]	Various pre-trained CNN models (Best: ResNet50)	Cvd.Acc: 96.1 (N,C-19), Cvd.Acc: 99.5(C-19,VP), Cvd.Acc: 99.7(C-19,BP)	N: 2800, C-19: 341, BP: 2772, VP: 1493		2
[184]	CNN model + SVM	Cvd.Acc (avg.): 95.81, Cvd. Prec(avg.): 95.27, F1 score(avg.): 94.94	N:1266 +317, C-19:460 + 116 P:3418 + 855 (Pvt.)		3
[185]	ResNet50+ SVM	Cvd.Sen:80, Cvd.Spe: 81, AUC: 0.81	Training and validation C-19:250, NC:250	Testing independent set C-19:74,NC:36 (Pvt.)	2
[186]	VisionPro Deep Learning™ + COGNEX’s	F-score: 95.3 (for segmented lung)	N: 7966+100,C-19: 258+100 P: 5451+100		3
[84]	Pillow library + HSGO + SVM	Cvd.Acc:99.65	C-19: 371, NC: 1341		2
[187]	CNN model	Cvd.Acc (avg.): 98.03, Cvd.Sen(avg.): 98.83, Cvd.Spe(avg.): 97	DS1:C-19: 217, NC: 1126 DS2:C-19: 2025, NC: 2025		2
[188]	AlexNet + Relief + SVM	Cvd.Acc: 99.18	N:1583, C-19: 219, P:4290		3
[189]	RGB to YUV and YUV to RGB + CNN	Cvd.Acc: 84.76, Cvd.Sen: 98.99, Cvd.Spe: 92.19, F-score: 0.9389,AUC: 0.5948	N:28,C-19:78,P: 79(each for BP and VP)		4
[190]	CNN model	Cvd.Acc: 98.44	Total: 392, C-19: 196		2
[191]	Deep CNN model	Cvd.Acc(avg.): 91.62, AUC:91.71	C-19 +ve: 538, C-19 –ve: 468		2
[192]	Deep CNN model	Cvd.Acc(avg.):99.2, Cvd.Sen(avg.):99.2,F1- score: 0.992	N, C-19: 2484 (each) N, C-19,P: 3829 (each)		2
		Cvd.Acc(avg.):95.2, Cvd.Sen(avg.):95.2,F1-score: 0.952			3
[193]	MobileNetV2	Cvd.Acc: 92.91, Cvd.Pre: 92	N: 234, C-19: 390		2
[49]	DenseNet201 model+ Quadratic SVM	Cvd.Acc: 98.16, Cvd.Sen: 98.93, Cvd.Spe: 98.77	N: 2924, C-19: 683,P: 4272		3
[194]	Cluster-based learning + Ensemble of classifiers	Cvd.Acc (avg.):100	N:79,C-19: 69, BP:79, VP:79		2(N,C-19)
		Cvd.Acc(avg.): 85.23			3(N,C-19,BP)
		Cvd.Acc(avg.): 74.05			4
[195]	Various deep CNN models are compared (Best: XCeptionNet)	F1-score: 0.97	N: 1345+238, C-19:490+ 86,P:3632+ 641 (Train + Test)		3
[196]	CNN model	Cvd.Acc: 98.19	N: 10,456, C-19: 573, P: 11,673 (Pvt.)		2(C-19,P)
		Cvd.Acc: 91.21			3
[197]	Federated learning model	Cvd.Acc: 98.72	N: 1266, C-19: 460,P: 3418 (Pvt.)		2(C-19,P)
		Cvd.Acc: 95.96			3
[80]	ResNet50 + ASSOA + MLP	Cvd.Acc: 99.70	Total: 5863		2(C-19+ve, C-19-ve)
[198]	Several CNN models are analyzed (Best: VGG16)	Cvd.Acc: 91	N:1341, C-19:219,P:1345		3
[199]	Semi-supervised open set domain adversarial network (SODA)	Avg. AUC-ROC Score: 0.9006(C-19), 0.9082(P)	With different domain target dataset		
[200]	VGG16 model	Cvd.Acc: 97, Cvd.Sen: 99, Cvd.Spe: 99, Cvd.Pre: 97, F-score: 98	N:1400, C-19: 210, P: 1400		3
[201]	CovFrameNet (deep learning architecture)	Cvd.Acc: 100, Cvd.Sen: 85, Cvd.Spe: 100, Cvd.Pre: 85, F-score: 90, AUC: 50	Using two different dataset		
[202]	Self-supervised super sample decomposition for transfer learning (4S-DT) model	Cvd.Acc: 97.54, Cvd.Sen: 97.88, Cvd.Spe: 97.15	DS1: N: 296, C-19: 388, SARS: 41		3(N, C-19, SARS)
		Cvd.Acc: 99.80, Cvd.Sen: 99.70, Cvd.Spe: 100	DS2: N: 1583,C-19: 576,P: 4273		3 (N,C-19,P)
[203]	VDI + Residual encoder + SVM	Cvd.Acc: 93.60, Cvd.Sen: 88, Cvd.Pre: 100, F1-score: 93.60	C-19: 315, NC: 357		2
[204]	RCoNet*^k^_s_*	Cvd.Acc (avg.):97.89, Cvd.Sen(avg.):97.76, Cvd.Spe(avg.):98.24, Cvd.PPV(avg.):97.93, F1-score(avg.):97.63	N: 8851, C-19: 238, P: 6045		3

Cvd.Acc (%): COVID accuracy, Cvd.Sen(%): COVID sensitivity, Cvd.Spe(%): COVID specificity, Cvd.Pre(%): COVID precision, Normal: N, COVID-19: C-19, Pneumonia: P, Bacterial pneumonia: BP, Viral pneumonia: VP, Tuberculosis: TB, Non-COVID: NC, Others: O, Abnormal: AB, Private: Pvt., DS: dataset, Severe: S, Non-severe: NS, Mild: M, Moderate: mod, Critical: cr, Infected/Infection: I, Not infected: NI, Community acquired pneumonia (CAP): P, Lung cancer: LC.

**Table 3:** State-of-the-art AI techniques to detect COVID-19 using CT scans.

Paper	Method Used: Preprocessing + Segmentation + Feature Extraction + Feature Selection + Classification or CNN + Classification		Result Obtained	Dataset (Most Are Public)	No. of Classes
[205]	Various deep models are analyzed (Best: ResNet101)		Cvd.Acc: 99.51, Cvd.Sen: 100, Cvd.Spe: 99.02, AUC: 0.994	C-19: 108,NC: 86,Total: 1020 slice, (Pvt.)	2
[206]	EfficientNet family based architecture		Cvd.Acc: 98.99, Cvd.Sen: 98.80, Cvd.PPV:99.20	DS 1- NC: 1230, C-19: 1252	2
			Cvd.Acc: 56.16, Cvd.Sen: 53.06, Cvd.PPV: 54.74 (Train DS 1 & Test DS2)	DS 2: NC: 463,C-19: 349	
[207]	LinkNet + DenseNet + DT		Cvd.Acc(avg.): 94.4, Cvd.Pre(avg.): 96.7, Cvd.Rec(avg.): 95.2, F1-score(avg.): 96.0	C-19:445,NC:233	2
[208]	novel conditional generative model, called CoSinGAN		Independent testing is done using 50 CT cases (for lung segmentation and infection learning)		
[93]	Intensity normalization and segmentation + Q-deformed entropy + ANOVA+ LSTM		Cvd.Acc: 99.68	N: 107,C-19: 118,P: 96	3
[209]	Modified Alexnet model		Cvd.Acc: 94.75, Cvd.Sen: 93.22, Cvd.Spe: 96.69, Cvd.PPV:97.27	C-19:3482,NC:2751 (Pvt.)	2
[210]	Ensemble various models using majority voting scheme		Cvd.Acc: 85.2, Cvd.Sen: 85.4, Cvd.Pre: 85.7,F-score: 0.852,AUC: 0.91	C-19 + ve: 349,C-19 -ve: 397	2
[211]	ResNet50		Cvd.Acc: 82.91, Cvd.Sen: 77.66, Cvd.Spe: 87.62	C-19:345,NC:397	2
[99]	CNN model with MODE		Cvd.Acc: outperforms competitive models by 1.9789%		2
[212]	Ensemble is built using ResNet152V2, DenseNet201, and VGG16		Cvd.Acc: 98.83, Cvd.Sen: 98.83, Cvd.Spe: 98.82,F-measure: 98.30,AUC: 98.28	N:3038,C-19:2373,P: 2890 TB: 3193	4
[36]	eXplainable Deep Learning approach (xDNN)		F1-score: 97.31	SARS-CoV-2: 1252 Non SARS-CoV-2: 1230	2
[35]	Multi-task and self-supervised learning		Cvd.Acc: 89, F1- score: 0.90, AUC: 0.98	C-19:349,NC: 463	2
[213]	Semi-Inf-Net		Cvd.Sen: 0.725, Cvd.Spe: 0.960, Dice: 0.739	100 images from 19 patients (Pvt)	C-19 lung Seg.
[214]	3D CNN model		Cvd.Acc: 87.50, Cvd.Sen: 86.90, Cvd.Spe: 90.10,F1-score: 82,AUC: 94.40	Train: 2186, Test: 2796 (Pvt.)	2 (CAP,C-19)
[215]	CNN model		Cvd.Acc (avg): 94.03, Cvd.Sen(avg.): 94.44, Cvd.Spe (avg.): 93.63	N: 320, C-19: 320 (Pvt.)	2
[92]	AlexNet + Guided WOA		Cvd.Acc: 87.50, AUC: 99.50	C-19: 334, NC-19: 794	2
[216]	Multi-task multi-slice deep learning system		Cvd.Acc: 95.21	N: 251,C-19: 245,H1N1: 105 CAP: 123 (Pvt.)	4
[217]	LBP and statistical features + ReliefF and NCA + DNN		Cvd.Acc: 95.84	N: 397,C-19: 349	2
[218]	Region growing + deep CNN model (ResNet101 as its backbone)		Cvd.Acc: 94.9	Total: 1110 patients with 5 classes	5
[219]	Radiomic features + mRMR + XGBoost		AUC: 0.95 ± 0.02	Total: 152 Patients	
[220]		Segmentation of infectious lung as ResNet50 backbone			
[221]	DTCT and GLCM + RF		Cvd.Acc (avg.): 72.2, Cvd.Sen(avg.): 77, Cvd.Spe(avg.): 68,AUROC (avg.): 0.8	C-19: 291, P: 279 (Pvt.)	2
[222]	ResGNet (Graphs are generated using ResNet101-C features)		Cvd.Acc (avg.): 96.62, Cvd.Sen(avg.): 97.33, Cvd.Spe(avg.): 95.91, Cvd.Pre(avg.): 96.21,F1-Score(avg.): 0.9665	N:148,C-19: 148 (Pvt.)	2
[223]	CNN model (DenseNet201) + ELM		Cvd.Acc: 98.36, Cvd.Sen: 98.28, Cvd.Spe: 98.44, Cvd.Pre: 98.22,F1-Score: 98.25, AUC: 98.36	C-19: 349,NC: 397	2
[224]	M ^2^ UNet (Multi-task multi-instance deep network)		Cvd.Acc (avg.): 98.5, Cvd.Sen(avg.): 95.2, Cvd.Pre(avg.): 97.5,F1-Score(avg.): 0.963 AUC(avg.): 0.991	S:51,NS: 191(Pvt.)	2
[225]	Dual-branch combination network (using UNet + ResNet50)		Cvd.Acc: 96.74, Cvd.Sen: 97.91, Cvd.Spe: 96.00,AUC: 0.9864	N: 75 scans, C-19: 48 scans (Pvt.)	2
[226]	Majority voting scheme with ResNet50		Cvd.Acc: 96, Cvd.Sen:100, Cvd.Spe: 96,AUC: 0.90	Two public datasets are used	2
[227]	HE + WF + AlexNet + SVM		Cvd.Acc: 96.69, Cvd.Sen: 96, Cvd.Spe: 98	N:500,C-19:488, P:500	3
[228]	DenseNet-201		Cvd.Acc: 97.8, Cvd.Sen: 98.1, Cvd.Spe: 97.3, Cvd.Pre: 98.4, F1-score: 98.25	C-19: 1500, NC: 1500	2
[229]	CLAHE + VGG-19 model		Cvd.Acc: 95.75, Cvd.Sen: 97.13,F1- score: 95.75, ROC-AUC: 99.30	C-19 +ve: 1252, C-19 -ve: 1230	2
[230]	VGG16 model and ensemble learning		Cvd.Acc: 93.57, Cvd.Sen: 94.21, Cvd.Spe: 93.93, Cvd.Pre: 89.4,F1-score: 91.74	N: 243,C-19: 790,P: 384	3
[61]	Z-score normalization and KF+CNN + fuzzy c-means + LDN		Cvd.Pre: 96, Cvd.Sen: 97, F-score: 97 and volume overlap error (VOE) of 5.6 ± 1:2%.		
[231]	Golden Key Tool + VGG model		Cvd.Acc: 100	DS1- N: 55, C-19: 349	2
			Cvd.Acc: 93.478, Cvd.Pre: 97.33, F1-score: 87.5	DS2- N: 55, C-19: 349, NC: 20	3
			Cvd.Acc: 90.12, Cvd.Pre: 90.6	DS3- C-19: 349, NC: 396	2
[232]	PatchShuffle Stochastic Pooling Neural Network (PSSPNN)		F1-score(avg.): 95.79	Total:521	4(N,C-19, P, TB)
[233]	Clinical information and chest CT features + XGBoost		Cvd.Sen: 90.91, Cvd.Spec: 97.96, AUC: 0.924	Total: 198	2 (M,S)
[234]	3D CU-Net		DSC: 0.960, 0.963, 0.771, Cvd.Sen: 0.969, 0.966, 0.837, Cvd.Spe: 0.998, 0.998, 0.998	C-19: 70 for detecting C-19 infection	
[235]	Tensor + COVID-19-Net (VGG16) + Transfer-Net (ResNet50)		Cvd.Acc: 94, Cvd.Sen: 96, Cvd.Spe: 92	N: 700, C-19: 700	2
[236]	Ensemble model (using Resnet18, Densenet201, Mobilenetv2 and Shufflenet)		Cvd.Acc: 96.51, Cvd.Sen: 96.96, Cvd.Spe: 96.00,F1-Score: 0.97,AUC: 0.99	C-19: 349,NC: 397	2
[237]	LungINFseg, model for segmentation		Cvd.Acc (avg.): 98.92, Cvd.Sen(avg.): 83.10, Cvd.Spe(avg.): 99.52, DSC(avg.):80.34 intersection over union (IoU) (avg.): 0.6877	20 labeled COVID-19 CT scans (1800 + annotated Slices)	
[238]	Feature Pyramid Network(FPN) DenseNet201 for detection		Cvd.Sen: 98.3 (m), Cvd.Sen: 71.2(mod), Cvd.Sen: 77.8(s), Cvd.Sen: 100(cr)	1110 subjects Severity classification	
[239]	Volume of interest based DenseNet-201		Cvd.Acc: 88.88, Cvd.Sen:89.77, Cvd.Spe: 94.73, F1-Score: 88.88	C-19: -moderate risk:40 severe risk:40 extreme risk:40	3
[240]	Various deep network architectures are analyzed using publicly available two COVID-19 CT datasets				2
[241]	UNet		F1-Score, improvement of 5.394 ± 3.015%.	+ve:492. -ve: 447	
[242]	Stationary wavelets + CNN model (Best: ResNet18)		Cvd.Acc: 99.4, Cvd.Sen: 100, Cvd.Spe: 98.6,AUC: 0.9965	C-19:349, NC:397	2
[243]	Gabor filter + convolution and pooling layers + RF		F1 score: 0.99	C-19: 349,NC: 397	2
[244]	Stacked autoencoder detector model		Cvd.Acc(avg.):94.7, Cvd.Sen(avg.):94.1, Cvd.Pre(avg.):96.54, F1-score (avg.):94.8	C-19: 275,NC: 195	2
[245]	DenseNet201 model + *k*-NN		Cvd.Acc, Cvd.Sen, Cvd.Pre, & F1-score:100	C-19:2740,Suspected Cases: 2740 (Private)	2
[246]	CNN model + MI and Relief-F and DA +SVM		Cvd.Acc: 98.39, Cvd.Sen: 97.78, Cvd.Pre: 98.21, F1-score: 0.98, AUC: 0.9952	SARS-CoV-2: 1252 Non SARS-CoV-2: 1230	2
			Cvd.Acc: 90.0, Cvd.Sen: 84.06, Cvd.Pre: 93.55,F1-score: 0.8855, AUC: 0.9414	C-19:349, NC: 463	
[247]	VGG19 model		Cvd.Acc: 94.52	C-19: 349,NC: 463	2
[248]	VGG16 model		Cvd.Acc: 98.0, Cvd.Sen: 99.0, Cvd.Spe: 94.9	N: 275, C-19: 195	2
[249]	Radiological features + Chi-square test + Ensemble classifier		Cvd.Acc: 91.94, Cvd.Sen: 93.54, Cvd.Spe: 90.32,AUC: 0.965	C-19: 306,non-COVID-19 pneumonia: 306 (Pvt.)	2
[250]	Various CNN and texture based approaches		Cvd.Acc (avg.): 95.99, Cvd.Sen(avg.): 94.04, Cvd.Spe(avg.): 99.01,F1-score(avg.): 0.9284, AUC (avg.): 0.9903	COVID-19: 386, NC: 1010	2
[251]	Worried deep neural network + pre-trained models (InceptionV3, ResNet50, and VGG19)		Cvd.Acc: 99.04, Cvd.Prec: 98.68, Cvd.Rec: 99.11,F-score: 98.90	Total: 2623 (Pvt.)	2(I,NI)
[252]	Density peak clustering approach		Structural similarity index (SSIM): 89	Total images: 12 (Pvt.)	C-19 Seg.
[253]	EfficientNet-b0 model		Cvd.Acc: 99.83, Cvd.Sen: 92.86, Cvd.Spe: 98.32, Cvd.PPV:91.92	Total images: 107,675 (Pvt.)	2(C-19,NC)
			Cvd.Acc: 97.32, Cvd.Sen: 99.71, Cvd.Spe: 95.98, Cvd.PPV: 93.26		2 (C-19,P)
[254]	EfficientNetB3		Cvd.Sen: 97.2, Cvd.Spe: 96.8,F1-score: 0.970, AUC: 0.997	N:105,C-19:143,P:147 (Pvt.)	3
			Cvd.Sen: 92.4, Cvd.Spe: 98.3,F1-score: 0.953,AUC: 0.989	N: 121,C-19: 119, P: 117(Pvt.)	3
			Cvd.Sen: 93.9, Cvd.Spe: 83.1,AUC: 0.954	C-19: 856,Non-P: 254 (Pvt.)	2
[255]	COVID Segnet		For COVID-19 segmentation: Dice Score: 0.726, Cvd.Sen.: 0.751, Cvd.Pre.: 0.726	Train: 731 Test: 130 patients (Pvt.)	Lung and infected regions seg.
			For lung segmentation: Dice Score: 0.987, Cvd.Sen.: 0.986, Cvd.Pre.: 0.990		
[256]	Anam-Net		Dice Score: 0.956, Cvd.Acc.: 98.5, Cvd.Sen.: 92.7, Cvd.Spe.: 99.8	N:929, AB:880	Anomalies seg.

**Table 4:** State-of-the-art AI techniques to detect COVID-19 using lung US imagery.

Paper	Method Used: Preprocessing + Segmentation + Feature Extraction + Feature Selection + Classification or CNN + Classification	Result Obtained	Dataset (Most Are Public)	No. of Classes
[257]	Features from various layers deep CNN model is fused	Cvd.Acc (avg.): 92.5, Cvd.Sen(avg.): 93.2, Cvd.Pre(avg.): 91.8	N: 53 + 15,C-19: 45+18,BP: 23 + 7	3
[258]	Autoencoder network and separable convolutional branches attached with a modified DenseNet201	17% more than the traditional DenseNet	Convex:38, Linear: 20 Score 0 (healthy) to Score 3 (worst-case)	4
[39]	Frame- and video-based CNN models (Best: VGG)	Cvd.Sen: 0.90 ± 0.08, Cvd.Spe: 0.96 ± 0.04	N: 90,C-19:92, BP: 73,VP: 6 (It includes videos and images)	3

**Table 5:** State-of-the-art AI techniques to detect COVID-19 using X-ray and CT scans.

Paper	Method Used: Preprocessing + Segmentation + Feature Extraction + Feature Selection + Classification or CNN + Classification	Result Obtained	Dataset (Most Are Public)	No. of Classes
[259]	VGG19 model	Cvd.Acc: 89.47, Cvd.Sen: 76.19, Cvd.Spe: 97.22	X-ray: 673 radiology images of 342 patients	2(N,C-19)
		Cvd.Acc: 95.61, Cvd.Sen: 96.55, Cvd.Spe: 95.29	SARS-CoV-2 CT: C-19:1252, NC: 1230	2(C-19,P)
		Cvd.Acc: 95, Cvd.Sen: 94.04, Cvd.Spe: 95.86	X-ray: 5856 images	2(C-19,NC)
[260]	VGG19 + CNN model	Cvd.Acc: 98.05, Cvd.Spe: 99.5, Cvd.Rec: 98.05, Cvd.Pre: 98.43, F1-Score: 98.24,AUC: 99.66	Total images: 33,676	4(N,C-19,P,LC)
[65]	LBP and MFrLFM + SFS	Cvd.Acc: 99.3±0.2, F1-score: 93.1±0.2, AUC: 94.9±0.1	Chest X-ray: 1926	2(C-19,NC)
		Cvd.Acc: 93.2±0.3, F1- score: 92.1±0.3,AUC: 93.2±0.3	CT scan: 2482	
[261]	COVID-ResNet53	Cvd.Acc: 97.1, Cvd.Sen: 98.9, Cvd.Spe: 95.7, Cvd.Pre: 94.5	X-ray: C-19: 4045, NC: 5500	2(C-19,NC)
		Cvd.Acc: 97.7, Cvd.Sen: 98.7, Cvd.Spe: 95.6, Cvd.Pre: 97.9	CT: C-19: 5427, NC: 2628	
[262]	CNN model	Cvd.Acc: 96.68, Cvd.Sen: 96.24, Cvd.Spe: 95.65	N: 7021,C-19: 1066, P:7021	3(N,C-19, P)
[263]	PF+ GraphCovidNet	Cvd.Acc, Cvd.Pre, Cvd.Sen,F1- score:100	SARS-CoV-2 CT N: 1229, C-19:1252	2
		Cvd.Acc, Cvd.Pre, Cvd.Sen,F1- score:100	CT: N: 407, C-19: 349	2
		Cvd.Acc, Cvd.Pre, Cvd.Sen,F1- score: 99.84	X-ray: N: 1592,C-19:504,P: 4343	3
[264]	HE and WF + Haralick texture feature and VGG16 model	Cvd.Acc: 93, Cvd.Sen: 90, Cvd.Pre: 91	N: 1349,C-19: 407,BP: 2538,VP: 1345	4
[265]	HE and WF + DenseNet103 + Haralick texture feature and ResNet101 model	Cvd.Acc: 94.9, Cvd. Sen: 93, Cvd. Pre: 93	Total images: 12,520, N: 4100, C-19: 220 P: 4100,Lung opacity: 4100	4
[266]	DenseNet121 + Bagging tree classifier	Cvd.Acc: 99	Total images: 274	2(N,C-19)
[267]	Contrastive multi-task convolutional neural network (CMT-CNN) CNN Model: EfficientNet	Cvd.Acc (avg.): 93.46, Cvd.Sen (avg.): 90.57, Cvd.Spe (avg.): 90.84 AUC (avg.): 89.33 (2-class)	CT scan: N: 1164,C-19: 1980,P:1614	2(C-19,O) 3(N,C-19,P)
		Cvd.Acc (avg.): 91.45 (3-class)		
		Cvd.Acc (avg.): 97.23, Cvd.Sen (avg.): 92.97, Cvd.Spe (avg.): 91.91 AUC (avg.): 92.13 (2-class)	X-ray: N: 1583, C-19: 231,P: 4007	
		Cvd.Acc (avg.): 93.49 (3-class)		
[268]	Contextual features reduced by convolutional filters (CFRCF)	Cvd.Acc: 94.23	CT: C-19: 349, NC: 397	2(C-19,NC)
			X-ray: C-19: 187, NC: 73	
[269]	CNN model	Cvd. Sen: 97.92, Cvd.Spe: 94.64, Cvd. Pre: 94.81,AUC: 0.9808	Total images: 672 (X-ray:336 and CT:336)	2(C-19,NC)
[270]	VGG16 + InceptionV3 models	Cvd.Sen: 100, Cvd.Pre: 0.97, F1: 0.98	CT: 746 X-ray: 268	2(N,C-19)
[271]	CovidNet model	Cvd. Acc: 100, Cvd. Sen: 100	CT: C-19: 1252, NC: 1230	2
		Cvd. Acc: 96.84, Cvd. Sen: 92.19	X-ray: N: 445, C-19:321, P:500	3
**Using all X-ray, CT, and US imageries**				
[272]	Pre-trained deep learning models: DenseNet-161, ResNet-34, VGG-16 and MobileNet-V2 are used	Cvd.Sen: 97.91, Cvd.Spe: 99.57, Cvd.Pre: 99.57,F1-score: 98.73	X-ray: C-19: 234, NC:234	2
		Cvd.Acc: 64.41, Cvd.Sen: 66.28, Cvd.Spe: 62.93, Cvd.Pre:58.67,F1-Score: 0.6225	CT: C-19: 392, NC:392	
		Cvd.Acc: 99.36, Cvd.Sen: 98.74, Cvd.Spe: 100, Cvd.Pre:100,F1-Score: 0.9973	US: C-19:19, NC:14	
[273]	VGG19 model	Cvd.Pre: 86	X-ray: N: 60,361,C-19:140,P:322	3
		Cvd.Pre: 84	CT: C-19: 349, NC: 397	2
		Cvd.Pre: 100	US: N: 235,C-19: 399,P: 277	3

**Table 6:** Average (Avg.) performance of COVID-19 detection systems.

**X-ray**					
**Class**	**Cvd.Acc (%)**	**Cvd.Sen (%)**	**Cvd.Spe (%)**	**F1-score (%)**	**AUC (%)**
2	97.05	95.37,086	94.79	96.11	95.45
3	94.78	95.63,542	97.10	85.71	93.55
4	91.69	94.335	97.16	83.32	64.74
5	92.41	82.96	95.24	81.49	88.1
**CT**					
**Class**	**Cvd.Acc (%)**	**Cvd.Sen (%)**	**Cvd.Spe (%)**	**F1-score (%)**	**AUC (%)**
2	92.99	92.61,897	93.28	94.57	91.40
3	94.55	95.016	95.55	92.08	99.3
4	97.02	98.83	98.82	97.9	98.28
5	--	--	--	--	94.9
**X-ray and CT**					
**Class**	**Cvd.Acc (%)**	**Cvd.Sen (%)**	**Cvd.Spe (%)**	**F1-score (%)**	**AUC (%)**
2	96.54	94.35	95.81	97.38	93.87
3	94.99	94.21	95.65	99.84	
4	95.52	94.75	--	98.24	99.66

## Data Availability

Not applicable.
